# Hormonal Medication Effects on Trichotillomania and Skin Picking Disorder

**DOI:** 10.3390/life16091434

**Published:** 2026-08-28

**Authors:** Sophia Boutouis, Megha Neelapu, Laurie Avila, Jon E. Grant

**Affiliations:** Department of Psychiatry & Behavioral Neuroscience, Pritzker School of Medicine, University of Chicago, Chicago, IL 60637, USA; sboutouis@uchicagomedicine.org (S.B.); megha.neelapu@bsd.uchicago.edu (M.N.); laurie.avila@bsd.uchicago.edu (L.A.)

**Keywords:** trichotillomania, hair pulling, skin picking, excoriation, hormones, birth control

## Abstract

**Background:** Trichotillomania (hair pulling disorder) and skin picking disorder often begin around puberty, suggesting the potential role of hormones in the etiology and maintenance of these conditions. However, little research has explored whether usage of hormonal medications, including birth control, affects symptoms in people with these disorders. **Methods:** Two hundred and ten women with self-reported trichotillomania and/or skin picking disorder were recruited from the community if they had previously taken a hormonal medication in their lifetime. Data were collected on the perceived relationship between hormonal medication use and severity of hair pulling or skin picking. **Results:** While some women reported positive and/or negative effects of hormonal medications on their hair pulling (18.0%) or skin picking (17.8%), most women (approximately 82.0%) reported no change in symptoms while taking these medications. **Conclusions:** Sex hormones may still influence the precipitation of trichotillomania and skin picking disorder; however, they appear to have continued effects on hair pulling and skin picking in adulthood for only a subset of those with these conditions.

## 1. Introduction

Trichotillomania and skin picking disorder are body-focused repetitive behaviors characterized by irresistible urges to pull one’s hair or pick one’s skin, often leading to noticeable hair loss or skin lesions [1]. While these conditions are relatively common (diagnosed in approximately 2% of the overall population), their pathophysiology remains poorly understood [2]. Compulsive hair pulling and skin picking often begin in adolescence [3,4], and both are more likely to be diagnosed in females than males during this developmental period [5,6]. While adolescence is also when many females begin to use birth control [7] or other hormonal medications (e.g., spironolactone for acne control), no research has explored whether hormonal medication use may be related to the etiology and maintenance of trichotillomania or skin picking disorder.

Several studies have suggested that sex hormones may be involved in trichotillomania, a view supported by its typical onset during puberty. In a validated animal model of trichotillomania, the administration of estradiol decreased the prevalence of barbering (a hair-plucking behavior in mice that mimics trichotillomania in humans) [8]. Similarly, in animal models of other compulsive behaviors, including marble burying and nesting, acute reduction in estrogen and progesterone by ovariectomy led to increased compulsive behavior, which was remedied by administering these hormones exogenously [9,10,11]. In a small sample of young females not on birth control (*n* = 11), Grant and Chamberlain analyzed levels of estradiol, progesterone, and testosterone in saliva samples and found that lower progesterone was associated with more severe hair pulling [12]. In the same sample, lower levels of all sex hormones were associated with worse overall psychosocial function.

In a sample of 56 women with trichotillomania, 20% of them reported fluctuations in their symptoms based on their menstrual cycle [13], and in another sample, 16 of 32 women (50%) reported the same [14]. Keuthen and colleagues examined the topic in greater detail and found that 53.3% of 45 women displayed more hair pulling during the premenstrual phase (i.e., the week before menstruation) [15]. A study regarding “focused” hair pulling detected a mild increase in “focused” pulling a few years prior to menopause, followed by a substantial decrease [16].

While these studies indicate that sex hormones may be involved in trichotillomania, no research has examined whether hormonal medication use is associated with hair pulling severity or associated outcomes. Additionally, neither one of these topics has been studied in skin picking disorder, a condition that has several phenomenological similarities and is highly comorbid with trichotillomania [17]. In related research regarding pregnancy, Keuthen and colleagues found no clear relationship between hair pulling and pregnancy [15]. These data were later corroborated by Grant and colleagues, who also found an unclear association between skin picking and pregnancy [18]. However, these data were collected retrospectively, and no biological measures were gathered. As such, the potential role of sex hormones in skin picking disorder remains unclear.

To fill crucial gaps in the literature, this exploratory study aimed to (1) examine the association between usage of hormonal medications, including birth control, and hair pulling and skin picking severity and (2) identify demographic and clinical variables associated with potential hormonal medication effects. Since hair pulling fluctuates during the menstrual cycle for some women [13,14], birth control may stabilize their hormone levels and reduce their symptoms. However, hormonal contraceptives can also negatively alter mood [19] and therefore exacerbate hair pulling or skin picking in some women. Given these opposing theories, we hoped to understand the effects of hormonal medications on trichotillomania and skin picking disorder to gain clarity on the pathophysiology of these disorders and help inform more effective treatments.

## 2. Methods

### 2.1. Participants

An online survey about the Sexual Health of People with Trichotillomania and Skin Picking Disorder was advertised in online Reddit support groups for trichotillomania, skin picking disorder, and other body-focused repetitive behaviors. A total of 639 women with self-reported trichotillomania and/or skin picking disorder were recruited from these groups. Participants were required to be between the ages of 18–65 and fluent in English to participate. Subjects were excluded if they did not review and sign the informed consent form, if they reported pulling their hair or picking their skin for less than 3 days per week or 15 min per day (indicates sporadic hair pulling/skin picking that does not meet DSM-5 or standard measure criteria for a body-focused repetitive behavior), if they did not indicate any distress or impairments resulting from their hair pulling/skin picking, if they completed the survey too quickly (i.e., in less than 3 min), or if they completed the survey more than once. After excluding participants for these reasons, 391 subjects remained in the sample. Of these, 212 reported taking hormonal medication(s) in their lifetime, but 2 did not provide information on how these medications affected their hair pulling or skin picking. Out of the 210 women in the final sample, 28.6% endorsed trichotillomania, 58.6% endorsed skin picking disorder, and 13.8% endorsed trichotillomania and skin picking disorder.

All participants were required to read and sign the IRB-approved informed consent form on REDCap via the eConsent Framework before proceeding with the survey. The form explained that participation in the survey was voluntary, and subjects were free to revoke their consent at any time. Participants could download a copy of the form following the consent procedures. If subjects consented to the survey, they were then taken to the questionnaires to be completed. The survey took approximately 30 min to complete. Participants who completed the survey could enter a raffle for a $100 virtual Visa gift card. Fifteen winners were randomly selected. Data collection occurred from April to May 2025. The Institutional Review Board of the University of Chicago approved the study and the consent statement (U of Chicago IRB #1 A BSD/UCMC IRB: IRB00000331; U of Chicago IRB #1B BSD/UCMC IRB: IRB00000735; U of Chicago IRB #1C BSD/UCMC IRB: IRB00002169; date of approval: 21 April 2025).

The authors assert that all procedures contributing to this work comply with the ethical standards of the relevant national and institutional committees on human experimentation and with the Helsinki Declaration of 1975, as revised in 2008.

### 2.2. Measures

The survey collected demographic information (i.e., age, sex, gender identity, race/ethnicity, education level, sexual orientation, occupational and relationship statuses) and self-reported psychiatric and pharmacological/psychotherapeutic intervention history. Participants were asked whether they have been diagnosed with any of the following psychiatric disorders in their lifetime: depression, any anxiety disorder (i.e., generalized anxiety disorder, social anxiety disorder, panic disorder, phobia), post-traumatic stress disorder (PTSD), attention-deficit/hyperactivity disorder (ADHD), obsessive–compulsive disorder (OCD), bipolar disorder, schizophrenia, any personality disorder, any eating disorder, alcohol use disorder, substance use disorder, or another disorder. Subjects could check as many options as applied to them, but only disorders that were endorsed by at least 5% of respondents were included in the analyses.

Participants were asked about their hair pulling and/or skin picking, particularly within the past week and at their worst during their lifetime. Subjects were prompted to type how many days per week and minutes or hours per day, on average, that they pull their hair or pick their skin. Participants were also asked to record common triggers for their hair pulling or skin picking and rate their perceived symptom-related distress and dysfunction on scales of 0 (“Not at all”) to 5 (“Very much”). The pre-selected options for triggers included “Stress or anxiety”, “Feeling down or irritable”, “Feeling overstimulated”, “Feeling happy or excited”, “Resting or relaxing”, “Boredom”, “Low stimulation activities (e.g., watching TV)”, “Work or school”, “Menstrual cycle”, “Diet”, “Texture or sight of hair or skin (e.g., split ends, bumps on skin)”, “Other”, “I have no particular triggers”, and “I don’t know”. Subjects could check which options applied to them.

Participants were then asked if they have ever taken a hormonal medication, including birth control. If so, they were prompted to write all the hormonal medications they have taken in their lifetime in a text box. Next, they responded to the multiple-choice question “Has taking any of these hormonal medications affected your hair pulling and/or skin picking?” Responses included “Yes, any or all of these medications made it better”, “Yes, any or all of these medications made it worse”, “Yes, sometimes the medications made it better, and sometimes it made it worse”, and “No, there was no effect”.

### 2.3. Statistical Analysis

Data from participants with self-reported trichotillomania (*n* = 60) and skin picking disorder (*n* = 123) were analyzed separately. However, subjects who endorsed both disorders (*n* = 29) were included in both analyses. Descriptive statistics for the sample were presented. Analyses of variance (ANOVAs) and chi-square tests were used to identify variables that were significantly associated with hormonal medication effects (i.e., no effects, positive effects, negative effects, mixed effects) on hair pulling and skin picking. All analyses and effect size calculations were conducted with IBM SPSS Statistics Version 31.0. Effect sizes were calculated in the forms of eta squared for ANOVAs (0.01 = small, 0.06 = medium, 0.14 = large) and Phi for chi-square tests (0.1 = small, 0.3 = medium, 0.5 = large). Statistical significance was set as *p* < 0.05 for all analyses as this was an exploratory study, and we aimed to detect smaller effects worthy of further exploration rather than to draw definitive conclusions.

## 3. Results

In total, 210 women with self-reported trichotillomania and/or skin picking disorder were included in the final sample. Of these, 206 indicated which hormonal medications they have taken in their lifetime (out of which 16 took two or more types of these medications in their lifetime). The vast majority (95.1%) of participants took a form of birth control, but some other subjects took spironolactone (3.9%; an aldosterone inhibitor often prescribed to treat hormonal acne) or testosterone (2.4%). Out of the 197 women who used birth control in their lifetime, 69.0% reported taking one form, whereas 31.0% reported taking two or more types. Additionally, 41.6% used a combination (estrogen and progestin) oral contraceptive, 9.1% used a progesterone/progestin-only oral contraceptive, 26.4% used a hormonal intrauterine device (IUD), 10.2% used a vaginal ring, 9.6% received an implant in their arm, 5.1% used an injectable form of birth control, 1.0% used emergency contraceptives, and 38.1% did not report which type of birth control that they used.

### 3.1. Trichotillomania

Eighty-nine participants in the sample self-identified as having active trichotillomania: 5.6% of these subjects said that their hormonal medications improved their pulling, 3.4% reported that they worsened their pulling, 9.0% indicated that they had mixed effects on their pulling, and 82.0% said that they had no effects on their pulling. The mean age of the trichotillomania sample was 30.69 (*SD* = 6.95) years. In the sample, 94.4% were cisgender women, and 5.6% were non-binary (assigned female at birth). In addition, 60.7% were heterosexual, 71.6% were non-Hispanic white, and 76.4% had a bachelor’s degree or higher.

Comorbid skin picking disorder was associated with medication effects, *X*^2^(3) = 8.208, *p* = 0.042, φ = 0.304 (medium effect size), such that those with both conditions were more likely to indicate mixed effects and less likely to report no effects. Education level was associated with the effects that hormonal medications had on pulling, *X*^2^(6) = 13.015, *p* = 0.043, φ = 0.382 (medium effect size). Those without a bachelor’s degree were more likely to say that their hormonal medications worsened or had mixed effects on their pulling (Table 1).

The number of days pulled in the past week was associated with the effects of hormonal medications on pulling, *F*(3) = 4.324, *p* = 0.007, η^2^ = 0.132 (medium effect size). Participants who indicated mixed or no effects pulled for the highest number of days in the past week, followed by those who reported positive effects. Those who said that there were negative effects pulled for the lowest number of days in the past week. The time per day pulled when pulling was at its worst was initially associated with the effects of hormonal medications on pulling, but after controlling for comorbid skin picking disorder using an analysis of covariance (ANCOVA), this relationship lost significance (*p* = 0.077). Menstrual cycle as a trigger was associated with the effects of hormonal medications on pulling, *X*^2^(3) = 11.545, *p* = 0.009, φ = 0.360 (medium effect size). Those who endorsed their menstrual cycle as a trigger for their pulling were more likely to indicate mixed effects and less likely to report no effects. Age of onset of pulling was not associated with the effects of hormonal medications on pulling (Table 2).

No common lifetime comorbidities were associated with the effects of hormonal medications on pulling (Table 3). Lifetime psychotropic medication use was associated with the effects of hormonal medications on pulling, *X*^2^(3) = 13.990, *p* = 0.003, φ = 0.396 (medium effect size). Those who reported lifetime psychotropic medication use were more likely to indicate negative effects of hormonal medications on their pulling and less likely to endorse no effects.

### 3.2. Skin Picking Disorder

In total, 152 participants in the sample self-identified as having active skin picking disorder: 4.6% said that their hormonal medications improved their picking, 3.3% reported that they worsened their picking, 9.9% indicated that they had mixed effects on their picking, and 82.2% said that they had no effects on their picking. The mean age of the skin picking disorder sample was 30.95 (*SD* = 8.11). In the sample, 89.5% were cisgender women, 3.3% were transgender men, and 7.2% were non-binary (assigned female at birth). In addition, 56.6% were heterosexual, 80.1% were non-Hispanic white, and 71.1% had a bachelor’s degree or higher.

Sexual orientation was associated with the effects of hormonal medications on picking, *X*^2^(3) = 8.093, *p* = 0.044, φ = 0.231 (small effect size), such that LGBTQ+ participants were more likely to say that their hormonal medications worsened or had mixed effects on their picking (Table 4).

The number of days picked in the past week was associated with the effects of hormonal medications on picking, *F*(3) = 3.014, *p* = 0.032, η^2^ = 0.058 (small effect size). Those who reported negative effects picked for the highest number of days in the past week, followed by those who said that there were positive or no effects. Those who endorsed mixed effects picked for the lowest number of days in the past week. Menstrual cycle as a picking trigger was also associated with the effects of hormonal medications on picking, *X*^2^(3) = 9.805, *p* = 0.020, φ = 0.254 (small effect size). Those who endorsed their menstrual cycle as a trigger for picking were more likely to indicate mixed effects and less likely to say that there were no effects (Table 5).

Finally, lifetime PTSD was associated with the effects of hormonal medications on picking, *X*^2^(3) = 10.902, *p* = 0.012, φ = 0.268 (small effect size). Those who endorsed lifetime PTSD were more likely to report mixed effects and less likely to say that there were no effects (Table 6).

## 4. Discussion

In our sample, approximately 1 in 5 of those with self-reported trichotillomania and/or skin picking disorder indicated that their hormonal medications impacted their hair pulling or skin picking. Previous research has suggested that sex hormones may influence the developmental course and presentation of trichotillomania, yet the specific nature of this link is still up for debate. This study proposes that while sex hormones could play a role in the precipitation of trichotillomania and skin picking disorder, they may only have continued effects into adulthood for some individuals with these disorders.

Several clinical and randomized placebo-controlled trials in people with psychiatric disorders have reported similar or mildly improved mood-related symptoms among those who use hormonal contraceptives compared to those who do not use them. However, there is a subset of individuals who develop depressive symptoms while using hormonal contraceptives [20]. The results of this study are similar, with most participants reporting no changes but some experiencing positive or negative changes in their hair pulling or skin picking while using hormonal medications. Perhaps the association between hormonal medication use and hair pulling/skin picking could have been mediated by depressive symptoms. This study aimed to add some clarity in this area of research for those with trichotillomania and skin picking disorder by identifying variables associated with the effects of hormonal medications on hair pulling and skin picking.

Participants who endorsed their menstrual cycle as a trigger for hair pulling or skin picking may have symptoms that are more sensitive to hormonal fluctuations, including those tied to hormonal medication use. Lower levels of sex hormones, which drop during menses, have been associated with increased hair pulling/skin picking in previous studies. One would then expect that those who reported more hair pulling/skin picking during menses would experience less pulling while taking hormonal medications, which raise sex hormone levels. Instead, they were more likely to report mixed effects. This suggests that the relationship between sex hormones and hair pulling/skin picking may not be uniform in direction across individuals, and that raising hormone levels may not straightforwardly reduce symptoms for everyone.

The education finding points in a similar direction. Those with a lower education level were also more likely to say that hormonal medications either had mixed effects on or worsened their pulling. While we did not directly measure socioeconomic status in this study, lower education may serve as a rough proxy for greater socioeconomic stress, which could interact with hormonal medication use. The relevant hormonal literature is itself mixed. Assari and colleagues found that financial difficulties were associated with higher levels of estradiol in female adolescents [21]. However, Bleil and colleagues found that higher neighborhood socioeconomic status predicted greater levels of sex hormone-binding globulin, which broadly indicates lower levels of sex hormones [22]. These findings do not point cleanly in one direction, which reinforces the possibility that the link between sex hormone levels and hair pulling is individual-specific rather than fixed. Overall, the results of this study call for further research on how changes in sex hormone levels affect hair pulling, and on whether that relationship differs across individuals.

Those who reported taking a psychotropic medication for trichotillomania in their lifetime were more likely to say that hormonal medications improved or worsened their pulling. Because both measures are lifetime and retrospective, we cannot establish which came first, so the following interpretations are tentative. One possibility is a treatment-seeking pattern: individuals who indicated that hormonal medications improved their pulling may have been more willing to try psychotropic medications for their pulling after observing a positive effect of medication use on their pulling. Simultaneously, those who indicated that hormonal medications worsened their pulling may have been more motivated to seek treatment in response to worsening symptoms. People who attend regular doctor’s appointments as part of receiving treatment are also encouraged to keep track of their symptoms and, as a result, may be more cognizant of whether their symptoms improve or worsen on certain medications.

Another potential explanation is a pharmacological interaction. Psychotropic and hormonal medications can interact, and some interactions may positively influence hair pulling symptoms, while other interactions may produce negative effects. There is limited research on the interactions between psychotropic medications and hormonal contraceptives [23]. Sex hormones, including hormonal contraceptives, can affect the pharmacokinetic processes of some psychotropic medications [23,24]. However, sex hormone levels in females tend to vary across the menstrual cycle and lifespan, which may be why observational studies regarding the effects of combined psychotropic medication and hormonal contraceptive use have highly variable results [20]. Therefore, an individual’s response to combined psychotropic medication and hormonal contraceptive use is difficult to predict, which may explain the findings of this study.

For participants with skin picking disorder, sexual orientation and a lifetime PTSD diagnosis were predictors of the effects of hormonal medications on picking. Non-heterosexual participants were more likely to say that their hormonal medications either worsened or had mixed effects on their picking. This exploratory finding rests on a small subgroup and should be interpreted cautiously until replicated. Some work suggests that non-heterosexual cisgender women may be exposed to higher levels of testosterone during the prenatal period, and these levels tend to persist into adulthood [25,26,27]. However, there is little evidence to suggest that non-heterosexual cisgender women significantly differ in levels of female sex hormones compared to heterosexual cisgender women, although more large-scales studies are needed [27]. Therefore, it is unlikely that inherent differences in female sex hormone levels between heterosexual and non-heterosexual cisgender women are the cause of this study’s finding. Another more probable explanation is that non-heterosexual individuals can experience minority stress, which interacts with hormonal medication use to produce worse symptoms [28]. In a large sample of female undergraduates, Masama and colleagues found that those using hormonal contraceptives reported more depressive symptoms and stress and had elevated plasma cortisol levels [29]. They proposed that hormonal contraceptives may increase sensitivity to various stressors, resulting in worse mental health outcomes. However, there are few high-quality studies on the effects of hormonal contraceptives on mood and other psychological correlates, so further research is needed in this area [30].

Participants with lifetime PTSD were more likely to report that their hormonal medications had mixed effects on their picking. Given that cisgender women are more likely than cisgender men to have anxiety, trauma-related, and stress-related disorders, researchers have hypothesized that sex hormones may play a role in the etiology and maintenance of these disorders. A literature review of this topic found that increased estradiol and progesterone can be protective but also increase vulnerability to anxiety, depending on the cognitive or behavioral processes occurring during the increase. For example, because of the effects of sex hormones on long-term memory processes, higher levels of progesterone during trauma may lead to over consolidation of traumatic memories, which can then lead to the development of PTSD [31]. Several previous studies have also found that traumatic experiences and PTSD are more common among those with skin picking disorder, and researchers have hypothesized that skin picking may be a coping mechanism for traumatic stress [32,33]. Therefore, improved or worsened PTSD symptoms associated with increased levels of sex hormones could also lead to changes in skin picking. The fact that unique predictors of the effects of hormonal medications on trichotillomania and skin picking disorder were found indicates that the pathways by which sex hormones affect these disorders may also differ. More research regarding how sex hormones influence skin picking is necessary.

While this study investigated a neglected research topic, namely how hormonal medications may influence hair pulling and skin picking, several limitations should be considered. The study relied on self-reported data and retrospective recall, which can involve bias and be inaccurate. Participants may have also incorrectly attributed hair pulling or skin picking symptom changes to hormonal medications when other factors were responsible. Although the number of participants is quite strong, it is still too small to thoroughly investigate differences between trichotillomania and skin picking, particularly when one includes the differences in hormonal medications and the retrospective nature of the data. For example, the role of PTSD as an associated variable to hormonal medication response in skin picking disorder, but not trichotillomania, demands further examination in larger studies. Importantly, the survey did not include a validated scale of trichotillomania or skin picking disorder, such as the Massachusetts General Hospital Hair Pulling or Skin Picking Scales. To keep the survey brief and reduce burden for participants, we asked them to simply rate their distress and impairments associated with hair pulling/skin picking. However, a longer, established measure would have captured these constructs better and verified subjects’ self-reported diagnoses. This may have resulted in the inclusion of individuals with subthreshold symptoms of trichotillomania or skin picking disorder. As such, the results of this study should be interpreted with caution and considered preliminary. Another limitation is that the sample was relatively small and lacking in racial/ethnic diversity (i.e., consisting largely of non-Hispanic white women). Additionally, the sample of those with trichotillomania included more highly educated and LGBTQ+ women compared to samples in other research [34]. Perhaps this is because our participants were recruited from online support groups, and they may not be representative of trichotillomania in medical or general population settings. There are other variables related to pulling and picking that we did not examine in this study, such as location on the body where participants pull and pick. For example, participants who pull or pick from the genital area may be more likely to report changes in their pulling or picking from fluctuations in sex hormone levels. We also did not examine potential differences in effects between types of birth control (e.g., combined estrogen and progestin versus progesterone/progestin-only oral contraceptives), as several participants in our sample (38.1%) did not know or report which form of birth control that they used. Future research should explore whether different hormonal medications have distinct effects on pulling and picking. Lastly, the triggers that we included in the survey primarily concerned emotional states, but there may be other environmental triggers that contributed to changes in hair pulling and skin picking.

Based on these findings, future work should examine hair pulling and skin picking at the time of hormonal medication use rather than retrospectively and record additional medication details such as dosage. This work could also include measures of hormonal pathways and stress to better inform mechanisms of fluctuations in symptoms and to better understand why experiences differ between individuals and between disorders.

## Figures and Tables

**Table 1:** Demographics for a sample of 89 women with self-reported trichotillomania, stratified by hormonal medication effects on hair pulling.

		Group							
		Total M (SD) or %	No Effects M (SD) or %	Positive Effects M (SD) or %	Negative Effects M (SD) or %	Mixed Effects M (SD) or %	*F*, Chi-Square	*p*	η^2^ or φ
All (*n* = 89)		100%	82.0%	5.6%	3.4%	9.0%			
Diagnosis (*n* = 89) *	TTM	66.3%	84.7%	6.8%	5.1%	3.4%	8.208	0.042	0.304
	TTM and SPD	33.7%	76.7%	3.3%	0%	20.0%			
Age (*n* = 89)		30.69 (6.95)	30.73 (6.69)	32.80 (12.81)	31.33 (7.51)	28.75 (5.44)	0.362	0.780	0.013
Sex and gender (*n* = 89)	Cisgender woman	94.4%	82.1%	6.0%	3.6%	8.3%	1.193	0.755	0.116
	Non-binary, AFAB	5.6%	80.0%	0%	0%	20.0%			
Ethnicity (*n* = 89)	Non-Hispanic/Latino	88.8%	83.5%	3.8%	2.5%	10.1%	6.828	0.078	0.277
	Hispanic/Latino	11.2%	70.0%	20.0%	10.0%	0%			
Race (*n* = 88)	White	78.4%	82.6%	5.8%	2.9%	8.7%			
	Asian	12.5%	72.7%	0%	9.1%	18.2%			
	Black	3.4%	100%	0%	0%	0%			
	Other	5.7%	80.0%	20.0%	0%	0%			
Sexual orientation (*n* = 89)	Heterosexual	60.7%	79.6%	9.3%	3.7%	7.4%	3.764	0.288	0.206
	Non-heterosexual	39.3%	85.7%	0%	2.9%	11.4%			
Employment status (*n* = 89)	Full-time	64.0%	87.7%	3.5%	1.8%	7.0%			
	Part-time	9.0%	100%	0%	0%	0%			
	Student	16.9%	66.7%	6.7%	6.7%	20.0%			
	Unemployed	5.6%	40.0%	20.0%	20.0%	20.0%			
	Other	4.5%	75.0%	25.0%	0%	0%			
Education (*n* = 89) *	Less than bachelor’s degree	23.6%	61.9%	4.8%	9.5%	23.8%	13.015	0.043	0.382
	Bachelor’s degree	46.1%	87.8%	4.9%	0%	7.3%			
	Master’s degree or higher	30.3%	88.9%	7.4%	3.7%	0%			
Relationship status (*n* = 89)	Married or engaged	38.2%	88.2%	8.8%	0%	2.9%	10.511	0.105	0.344
	In a relationship	30.3%	85.2%	3.7%	0%	11.1%			
	Single	31.5%	71.4%	3.6%	10.7%	14.3%			

Note. * indicates a statistically significant difference between groups (*p* < 0.05), Chi-square tests were not performed with respect to race and employment status due to expected cell counts of less than 5. TTM = trichotillomania, SPD = skin picking disorder, AFAB = assigned female at birth.

**Table 2:** Hair pulling details for a sample of 89 women with self-reported trichotillomania, stratified by hormonal medication effects on hair pulling.

			Group							
			Total M (SD) or %	No Effects M (SD) or %	Positive Effects M (SD) or %	Negative Effects M (SD) or %	Mixed Effects M (SD) or %	*F*, Chi-Square	*p*	η^2^ or φ
Age of onset (*n* = 89)			13.60 (5.11)	13.52 (5.15)	12.80 (3.27)	11.67 (6.43)	15.50 (5.58)	0.551	0.649	0.019
Pulling in the past week (*n* = 89)	Number of days pulled **		6.00 (1.42)	6.12 (1.37)	5.00 (1.22)	3.67 (1.15)	6.38 (1.19)	4.324	0.007	0.132
	Time per day pulled, minutes		89.35 (104.42)	91.05 (110.89)	72.00 (44.24)	25.00 (5.00)	108.75 (83.74)	0.516	0.673	0.018
	How much pulling caused distress (0–5)		3.74 (1.25)	3.78 (1.23)	3.00 (1.87)	3.33 (1.15)	4.00 (1.07)	0.829	0.482	0.028
	How much pulling interfered with daily life (0–5)		2.84 (1.37)	2.81 (1.40)	3.00 (1.00)	2.33 (2.08)	3.25 (1.16)	0.401	0.752	0.014
Pulling at its worst in lifetime	Number of days pulled (*n* = 89)		6.70 (0.79)	6.73 (0.75)	6.00 (1.41)	7.00 (0.00)	6.75 (0.71)	1.519	0.216	0.051
	Time per day pulled, minutes (*n* = 88)	Uncontrolled *	120.48 (138.83)	112.56 (119.00)	125.00 (57.23)	340.00 (484.97)	105.71 (61.88)	2.766	0.047	0.090
		Controlled for comorbid SPD	N/A	111.76	117.81	321.72	127.06	2.368	0.077	0.079
	How much pulling caused distress (0–5) (*n* = 89)		4.37 (0.93)	4.38 (0.86)	4.00 (1.73)	5.00 (0.00)	4.25 (1.16)	0.759	0.520	0.026
	How much pulling interfered with daily life (0–5) (*n* = 89)		3.61 (1.22)	3.51 (1.24)	4.00 (0.71)	4.67 (0.58)	3.88 (1.36)	1.227	0.305	0.042
Major triggers (*n* = 89)	Stress or anxiety	Yes	94.4%	81.0%	6.0%	3.6%	9.5%	1.161	0.762	0.114
		No	5.6%	100%	0%	0%	0%			
	Feeling down or irritable	Yes	55.1%	75.5%	10.2%	2.0%	12.2%	6.503	0.090	0.270
		No	44.9%	90.0%	0%	5.0%	5.0%			
	Feeling overstimulated	Yes	60.7%	77.8%	5.6%	5.6%	11.1%	2.935	0.402	0.182
		No	39.3%	88.6%	5.7%	0%	5.7%			
	Resting or relaxing	Yes	73.0%	84.6%	4.6%	3.1%	7.7%	1.141	0.767	0.113
		No	27.0%	75.0%	8.3%	4.2%	12.5%			
	Boredom	Yes	91.0%	82.7%	4.9%	2.5%	9.9%	3.757	0.289	0.205
		No	9.0%	75.0%	12.5%	12.5%	0%			
	Low stimulation activities	Yes	86.5%	81.8%	5.2%	2.6%	10.4%	2.445	0.485	0.166
		No	13.5%	83.3%	8.3%	8.3%	0%			
	Work or school	Yes	55.1%	81.6%	6.1%	4.1%	8.2%	0.297	0.960	0.058
		No	44.9%	82.5%	5.0%	2.5%	10.0%			
	Menstrual cycle **	Yes	20.2%	55.6%	11.1%	11.1%	22.2%	11.545	0.009	0.360
		No	79.8%	88.7%	4.2%	1.4%	5.6%			
	Texture or sight of hair	Yes	87.6%	82.1%	3.8%	3.8%	10.3%	5.078	0.166	0.239
		No	12.4%	81.8%	18.2%	0%	0%			

Note. * indicates a statistically significant difference between groups (*p* < 0.05), ** indicates a statistically significant difference between groups (*p* < 0.01).

**Table 3:** Major lifetime comorbidities and treatment history for a sample of 89 women with self-reported trichotillomania, stratified by hormonal medication effects on hair pulling.

			Group							
			Total M (SD) or %	No Effects M (SD) or %	Positive Effects M (SD) or %	Negative Effects M (SD) or %	Mixed Effects M (SD) or %	Chi-Square	*p*	φ
Lifetime comorbidities (*n* = 88)	Any	Yes	90.9%	81.3%	6.3%	3.8%	8.8%	0.947	0.814	0.104
		No	9.1%	87.5%	0%	0%	12.5%			
	Depression	Yes	68.2%	78.3%	6.7%	5.0%	10.0%	2.173	0.537	0.157
		No	31.8%	89.3%	3.6%	0%	7.1%			
	Anxiety	Yes	77.3%	82.4%	5.9%	2.9%	8.8%	0.247	0.970	0.053
		No	22.7%	80.0%	5.0%	5.0%	10.0%			
	PTSD	Yes	22.7%	65.0%	10.0%	10.0%	15.0%	6.036	0.110	0.262
		No	77.3%	86.8%	4.4%	1.5%	7.4%			
	ADHD	Yes	39.8%	80.0%	2.9%	2.9%	14.3%	2.617	0.455	0.172
		No	60.2%	83.0%	7.5%	3.8%	5.7%			
	OCD	Yes	18.2%	68.8%	12.5%	12.5%	6.3%	6.923	0.074	0.280
		No	81.8%	84.7%	4.2%	1.4%	9.7%			
Lifetime treatment for pulling (*n* = 89)	Any	Yes	48.3%	74.4%	9.3%	7.0%	9.3%	5.815	0.121	0.256
		No	51.7%	89.1%	2.2%	0%	8.7%			
	Medication **	Yes	20.2%	66.7%	11.1%	16.7%	5.6%	13.990	0.003	0.396
		No	79.8%	85.9%	4.2%	0%	9.9%			
	Therapy	Yes	37.5%	75.8%	12.1%	3.0%	9.1%	4.113	0.250	0.216
		No	62.5%	85.5%	1.8%	3.6%	9.1%			

Note. ** indicates a statistically significant difference between groups (*p* < 0.01). PTSD = post-traumatic stress disorder, ADHD = attention-deficit/hyperactivity disorder, OCD = obsessive–compulsive disorder.

**Table 4:** Demographics for a sample of 152 women with self-reported skin picking disorder, stratified by hormonal medication effects on skin picking.

		Group							
		Total M (SD) or %	No Effects M (SD) or %	Positive Effects M (SD) or %	Negative Effects M (SD) or %	Mixed Effects M (SD) or %	*F*, Chi-Square	*p*	η^2^ or φ
All (*n* = 152)		100%	82.2%	4.6%	3.3%	9.9%	N/A	N/A	N/A
Diagnosis (*n* = 152)	SPD	80.3%	82.8%	4.9%	4.1%	8.2%	3.134	0.371	0.144
	SPD and TTM	19.7%	80.0%	3.3%	0%	16.7%			
Age (*n* = 152)		30.95 (8.11)	31.22 (8.34)	30.14 (10.54)	30.80 (7.01)	29.13 (5.19)	0.318	0.812	0.006
Sex and gender (*n* = 152)	Cisgender woman	89.5%	83.1%	4.4%	2.2%	10.3%	11.818	0.066	0.279
	Non-binary, AFAB	7.2%	72.7%	0%	18.2%	9.1%			
	Transgender man	3.3%	80.0%	20.0%	0%	0%			
Ethnicity (*n* = 152)	Non-Hispanic/Latino	92.8%	81.6%	5.0%	3.5%	9.9%	1.052	0.789	0.083
	Hispanic/Latino	7.2%	90.9%	0%	0%	9.1%			
Race (*n* = 151)	White	84.8%	80.5%	5.5%	3.1%	10.9%	3.738	0.928	0.157
	Asian	9.3%	85.7%	0%	7.1%	7.1%			
	Black	2.0%	100%	0%	0%	0%			
	Other	4.0%	100%	0%	0%	0%			
Sexual orientation (*n* = 152) *	Heterosexual	56.6%	86.0%	5.8%	0%	8.1%	8.093	0.044	0.231
	Non-heterosexual	43.4%	77.3%	3.0%	7.6%	12.1%			
Employment status (*n* = 152)	Full-time	63.8%	86.6%	2.1%	2.1%	9.3%	15.367	0.222	0.318
	Part-time	7.2%	90.9%	9.1%	0%	0%			
	Student	15.8%	75.0%	8.3%	8.3%	8.3%			
	Unemployed	10.5%	56.3%	12.5%	6.3%	25.0%			
	Other	2.6%	100%	0%	0%	0%			
Education (*n* = 152)	Less than bachelor’s degree	28.9%	77.3%	4.5%	4.5%	13.6%	6.151	0.406	0.201
	Bachelor’s degree	39.5%	83.3%	6.7%	0%	10.0%			
	Master’s degree or higher	31.6%	85.4%	2.1%	6.3%	6.3%			
Relationship status (*n* = 151)	Married or engaged	32.5%	83.7%	4.1%	0%	12.2%	3.462	0.749	0.151
	In a relationship	33.1%	82.0%	4.0%	6.0%	8.0%			
	Single	34.4%	80.8%	5.8%	3.8%	9.6%			

Note. * indicates a statistically significant difference between groups (*p* < 0.05). SPD = skin picking disorder, TTM = trichotillomania, AFAB = assigned female at birth.

**Table 5:** Skin picking details for a sample of 152 women with self-reported skin picking disorder, stratified by hormonal medication effects on skin picking.

			Group							
			Total M (SD) or %	No Effects M (SD) or %	Positive Effects M (SD) or %	Negative Effects M (SD) or %	Mixed Effects M (SD) or %	*F*, Chi-Square	*p*	η^2^ or φ
Age of onset (*n* = 152)			10.14 (6.60)	9.98 (6.53)	13.14 (12.08)	6.60 (3.36)	11.20 (3.99)	1.118	0.344	0.022
Picking in the past week (*n* = 152)	Number of days picked *		6.34 (1.21)	6.42 (1.14)	6.29 (1.25)	7.00 (0.00)	5.53 (1.60)	3.014	0.032	0.058
	Time per day picked, minutes		75.23 (88.83)	74.76 (93.07)	73.57 (59.77)	71.00 (35.07)	81.33 (80.34)	0.029	0.993	0.001
	How much picking caused distress (0–5)		3.94 (1.11)	3.93 (1.12)	3.71 (0.95)	4.60 (0.55)	3.93 (1.22)	0.692	0.558	0.014
	How much picking interfered with daily life (0–5)		2.93 (1.32)	2.83 (1.34)	3.71 (0.95)	3.80 (0.84)	3.13 (1.19)	1.944	0.125	0.038
Picking at its worst in lifetime	Number of days picked (*n* = 152)		6.77 (0.70)	6.73 (0.76)	7.00 (0.00)	7.00 (0.00)	6.93 (0.26)	0.864	0.461	0.017
	Time per day picked, minutes (*n* = 152)		115.40 (125.55)	111.45 (126.90)	72.86 (23.60)	168.00 (113.67)	150.67 (141.70)	0.996	0.396	0.020
	How much picking caused distress (0–5) (*n* = 152)		4.49 (0.78)	4.45 (0.82)	4.71 (0.49)	4.80 (0.45)	4.60 (0.63)	0.670	0.571	0.013
	How much picking interfered with daily life (0–5) (*n* = 151)		3.74 (1.22)	3.62 (1.26)	4.14 (0.90)	4.80 (0.45)	4.13 (0.92)	2.496	0.062	0.048
Major triggers (*n* = 152)	Stress or anxiety	Yes	89.5%	81.6%	5.1%	2.9%	10.3%	1.597	0.680	0.103
		No	10.5%	87.5%	0%	6.3%	6.3%			
	Feeling down or irritable	Yes	60.5%	80.4%	6.5%	3.3%	9.8%	1.953	0.582	0.113
		No	39.5%	85.0%	1.7%	3.3%	10.0%			
	Feeling overstimulated	Yes	62.5%	78.9%	5.3%	4.2%	11.6%	1.976	0.577	0.114
		No	37.5%	87.7%	3.5%	1.8%	7.0%			
	Resting or relaxing	Yes	53.9%	82.9%	4.9%	2.4%	9.8%	0.433	0.933	0.053
		No	46.1%	81.4%	4.3%	4.3%	10.0%			
	Boredom	Yes	77.0%	83.8%	5.1%	2.6%	8.5%	2.157	0.540	0.119
		No	23.0%	77.1%	2.9%	5.7%	14.3%			
	Low stimulation activities	Yes	74.3%	84.1%	4.4%	2.7%	8.8%	1.214	0.750	0.089
		No	25.7%	76.9%	5.1%	5.1%	12.8%			
	Work or school	Yes	48.7%	82.4%	6.8%	4.1%	6.8%	3.121	0.373	0.143
		No	51.3%	82.1%	2.6%	2.6%	12.8%			
	Menstrual cycle *	Yes	15.1%	60.9%	8.7%	4.3%	26.1%	9.805	0.020	0.254
		No	84.9%	86.0%	3.9%	3.1%	7.0%			
	Texture or sight of skin	Yes	95.4%	82.1%	4.8%	3.4%	9.7%	0.735	0.865	0.070
		No	4.6%	85.7%	0%	0%	14.3%			

Note. * indicates a statistically significant difference between groups (*p* < 0.05).

**Table 6:** Major lifetime comorbidities and treatment history for a sample of 152 women with self-reported skin picking disorder, stratified by hormonal medication effects on skin picking.

			Group							
			Total M (SD) or %	No Effects M (SD) or %	Positive Effects M (SD) or %	Negative Effects M (SD) or %	Mixed Effects M (SD) or %	Chi-Square	*p*	φ
Lifetime comorbidities (*n* = 152)	Any	Yes	86.8%	81.8%	4.5%	3.8%	9.8%	0.787	0.853	0.072
		No	13.2%	85.0%	5.0%	0%	10.0%			
	Depression	Yes	65.8%	81.0%	6.0%	4.0%	9.0%	1.961	0.581	0.114
		No	34.2%	84.6%	1.9%	1.9%	11.5%			
	Anxiety	Yes	75.0%	80.7%	4.4%	3.5%	11.4%	1.334	0.721	0.094
		No	25.0%	86.8%	5.3%	2.6%	5.3%			
	PTSD *	Yes	21.7%	72.7%	3.0%	0%	24.2%	10.902	0.012	0.268
		No	78.3%	84.9%	5.0%	4.2%	5.9%			
	ADHD	Yes	45.4%	79.7%	1.4%	4.3%	14.5%	6.000	0.112	0.199
		No	54.6%	84.3%	7.2%	2.4%	6.0%			
	OCD	Yes	20.4%	74.2%	0%	6.5%	19.4%	6.853	0.077	0.212
		No	79.6%	84.3%	5.8%	2.5%	7.4%			
Lifetime treatment for picking (*n* = 152)	Any	Yes	27.6%	85.7%	0%	2.4%	11.9%	1.366	0.714	0.095
		No	72.4%	80.9%	6.4%	3.6%	9.1%			
	Medication	Yes	13.2%	80.0%	0%	5.0%	15.0%	1.876	0.599	0.111
		No	86.8%	82.4%	5.3%	3.1%	9.2%			
	Therapy	Yes	21.7%	84.8%	0%	3.0%	12.1%	2.202	0.532	0.120
		No	78.3%	81.5%	5.9%	3.4%	9.2%			

Note. * indicates a statistically significant difference between groups (*p* < 0.05). PTSD = post-traumatic stress disorder, ADHD = attention-deficit/hyperactivity disorder, OCD = obsessive–compulsive disorder.

## Data Availability

Data is available upon request.

## References

[1] American Psychiatric Association (2013). Obsessive-compulsive and related disorders. Diagnostic and Statistical Manual of Mental Disorders.

[2] Grant J.E., Chamberlain S.R. (2021). Trichotillomania and skin-picking disorder: An update. Focus.

[3] Ricketts E.J., Snorrason Í., Kircanski K., Alexander J.R., Thamrin H., Flessner C.A., Franklin M.E., Piacentini J., Woods D.W. (2018). A latent profile analysis of age of onset in pathological skin picking. Compr. Psychiatry.

[4] Ricketts E.J., Snorrason I., Kircanski K., Alexander J.R., Stiede J.T., Thamrin H., Flessner C.A., Franklin M.E., Keuthen N.J., Walther M.R. (2019). A latent profile analysis of age of onset in trichotillomania. Ann. Clin. Psychiatry.

[5] Grant J.E., Chamberlain S.R. (2016). Trichotillomania. Am. J. Psychiatry.

[6] Grant J.E., Chamberlain S.R. (2022). Characteristics of 262 adults with skin picking disorder. Compr. Psychiatry.

[7] Daniels K., Abma J.C. (2018). Current Contraceptive Status Among Women Aged 15–49: United States, 2015–2017.

[8] Husk S.E. (2010). The Role of Estrogen in the Onset and Performance of Plucking Behavior in a Mouse Model of Trichotillomania. Master’s Thesis.

[9] Fernández-Guasti A., Agrati D., Reyes R., Ferreira A. (2006). Ovarian steroids counteract serotonergic drugs actions in an animal model of obsessive-compulsive disorder. Psychoneuroendocrinology.

[10] Flaisher-Grinberg S., Albelda N., Gitter L., Weltman K., Arad M., Joel D. (2009). Ovarian hormones modulate ‘compulsive’ lever-pressing in female rats. Horm. Behav..

[11] Mitra S., Bastos C.P., Bates K., Pereira G.S., Bult-Ito A. (2016). Ovarian sex hormones modulate compulsive, affective and cognitive functions in a non-induced mouse model of obsessive-compulsive disorder. Front. Behav. Neurosci..

[12] Grant J.E., Chamberlain S.R. (2018). Salivary sex hormones in adolescent females with trichotillomania. Psychiatry Res..

[13] Christenson G.A., Mackenzie T.B., Mitchell J.E. (1991). Characteristics of 60 adult chronic hair pullers. Am. J. Psychiatry.

[14] Lochner C., Seedat S., du Toit P.L., Nel D.G., Niehaus D.J., Sandler R., Stein D.J. (2005). Obsessive-compulsive disorder and trichotillomania: A phenomenological comparison. BMC Psychiatry.

[15] Keuthen N.J., O’Sullivan R.L., Hayday C.F., Peets K.E., Jenike M.A., Baer L. (1997). The relationship of menstrual cycle and pregnancy to compulsive hairpulling. Psychother. Psychosom..

[16] Flessner C.A., Woods D.W., Franklin M.E., Keuthen N.J., Piacentini J. (2008). Cross-sectional study of women with trichotillomania: A preliminary examination of pulling styles, severity, phenomenology, and functional impact. Child Psychiatry Hum. Dev..

[17] Lin A., Farhat L.C., Flores J.M., Levine J.L.S., Fernandez T.V., Bloch M.H., Olfson E. (2023). Characteristics of trichotillomania and excoriation disorder across the lifespan. Psychiatry Res..

[18] Grant J.E., Neelapu M., Avila L., Boutouis S., Chamberlain S.R. (2026). Trichotillomania, skin picking disorder, and pregnancy. Compr. Psychiatry.

[19] Brás B.P., Silva J.F., Caldas F., Almeida H.S. (2025). The burden of birth control: A narrative review on the mood-related side effects of hormonal contraception. Eur. Psychiatry.

[20] McCloskey L.R., Wisner K.L., Cattan M.K., Betcher H.K., Stika C.S., Kiley J.W. (2021). Contraception for women with psychiatric disorders. Am. J. Psychiatry.

[21] Assari S., Boyce S., Bazargan M., Caldwell C.H. (2020). Race, socioeconomic status, and sex hormones among male and female American adolescents. Reprod. Med..

[22] Bleil M.E., Appelhans B.M., Latham M.D., Irving M.A., Gregorich S.E., Adler N.E., Cedars M.I. (2015). Neighborhood socioeconomic status during childhood versus puberty in relation to endogenous sex hormone levels in adult women. Nurs. Res..

[23] Berry-Bibee E.N., Kim M.-J., Simmons K.B., Tepper N.K., Riley H.E.M., Pagano H.P., Curtis K.M. (2016). Drug interactions between hormonal contraceptives and psychotropic drugs: A systematic review. Contraception.

[24] Damoiseaux V.A., Proost J.H., Jiawan V.C., Melgert B.N. (2014). Sex differences in the pharmacokinetics of antidepressants: Influence of female sex hormones and oral contraceptives. Clin. Pharmacokinet..

[25] Balthazart J. (2011). Minireview: Hormones and human sexual orientation. Endocrinology.

[26] Roselli C.E. (2018). Neurobiology of gender identity and sexual orientation. J. Neuroendocrinol..

[27] Harris A., Bewley S., Meads C. (2020). Sex hormone levels in lesbian, bisexual, and heterosexual women: Systematic review and exploratory meta-analysis. Arch. Sex. Behav..

[28] Meyer I.H. (2003). Prejudice, social stress, and mental health in lesbian, gay, and bisexual populations: Conceptual issues and research evidence. Psychol. Bull..

[29] Masama C., Jarkas D.A., Thaw E., Daneshmend A.Z.B., Franklyn S.I., Beaurepaire C., McQuaid R.J. (2022). Hormone contraceptive use in young women: Altered mood states, neuroendocrine and inflammatory biomarkers. Horm. Behav..

[30] Lewis C.A., Kimmig A.-C.S., Zsido R.G., Jank A., Derntl B., Sacher J. (2019). Effects of hormonal contraceptives on mood: A focus on emotion recognition and reactivity, reward processing, and stress response. Curr. Psychiatry Rep..

[31] Li S.H., Graham B.M. (2017). Why are women so vulnerable to anxiety, trauma-related and stress-related disorders? The potential role of sex hormones. Lancet Psychiatry.

[32] Özten E., Hızlı Sayar G., Kağan G., Işık S., Karamustafalıoğlu O., Eryilmaz G. (2015). The relationship of psychological trauma with trichotillomania and skin picking. Neuropsychiatr. Dis. Treat..

[33] Grant J.E., Chamberlain S.R. (2020). Prevalence of skin picking (excoriation) disorder. J. Psychiatr. Res..

[34] Grant J., Collins M., Chamberlain S., Ehsan D. (2023). Trichotillomania in sexual minority individuals. Ann. Clin. Psychiatry.

